# Can Concurrent Fibrate Use Reduce Cardiovascular Risks among Moderate Chronic Kidney Disease Patients Undergoing Statin Therapy? A Cohort Study

**DOI:** 10.3390/jcm13010168

**Published:** 2023-12-28

**Authors:** Li-Yi Ma, Pei-Chun Fan, Chao-Yu Chen, Yi-Ran Tu, Ching-Chung Hsiao, Chieh-Li Yen, Chih-Hsiang Chang

**Affiliations:** 1 Kidney Research Center, Department of Nephrology, Chang Gung Memorial Hospital, Linkou Branch, Taoyuan 333423, Taiwan; maliyi24@gmail.com (L.-Y.M.); franwis1023@gmail.com (P.-C.F.); chaoyuclaire@gmail.com (C.-Y.C.); yirantu1020@gmail.com (Y.-R.T.); 2 College of Medicine, Chang Gung University, Taoyuan 333323, Taiwan; colinhua0123@gmail.com; 3 Department of Nephrology, New Taipei Municipal Tucheng Hospital, New Taipei 236017, Taiwan

**Keywords:** hypertriglyceridemia, TG, chronic kidney disease, CKD, fibrate, AMI, MACCEs

## Abstract

The role of fibrates in treating hypertriglyceridemia in chronic kidney disease (CKD) patients to prevent cardiovascular disease (CVD) has been insufficiently investigated. Since statin is considered the first-line treatment for dyslipidemia in CKD patients, this study aims to evaluate the role of concurrent fibrate therapy with statins among moderate CKD patients. We recruited CKD3 patients from the Chang Gung Research Database who were receiving statin treatment but had not previously been administered ezetimibe or niacin. The participants were divided into two groups based on their use of fibrates (fibrate group) or those with triglyceride levels >200 mg/dL without fibrate treatment (non-fibrate group). The fibrate group (*n* = 954) only exhibited a significantly lower incidence of AMI (4.4% vs. 5.4%, HR: 0.77, 95% CI: 0.61 to 0.98). The risk of major adverse cardiovascular and cerebrovascular events (14.7% vs. 15.6%, HR: 0.91, 95% CI: 0.72 to 1.15) and all-cause mortality (5.7% vs. 6.1%, HR: 0.91, 95% CI: 0.63 to 1.30) did not significantly differ between the fibrate group and the non-fibrate group (*n* = 2358). In moderate CKD patients, combining fibrate therapy with statins may not offer additional cardiovascular protection compared to statin alone.

## 1. Introduction

Dyslipidemia has become a global health concern because the prevalence of dyslipidemia is now increasing not only in high-income settings but even in low-income settings because of dietary and behavioral changes throughout the world. Elevated plasma low-density lipoprotein cholesterol (LDL-C) levels are a major causal factor for ischemic heart disease (IHD) and ischemic stroke in both the developed and the developing world [1]. In addition, elevated triglycerides (TGs) level (≥1.7 mmol/L or ≥150 mg/dL), low plasma high-density lipoprotein cholesterol (HDL-C) level (<1.03 mmol/L for men or <1.29 mmol/L for women), and high plasma small dense low-density lipoprotein cholesterol (sdLDL-C) level are a condition referred to as atherogenic dyslipidemia, which indicates the presence of metabolic diseases and is associated with overweight, obesity, or diabetes mellitus [1,2].

Chronic kidney disease (CKD) has also been another worldwide health concern [3]. CKD has a strong association with dyslipidemia, which can affect kidney function and increase the risk for cardiovascular disease (CVD) development [4]. Among patients with CKD, the leading cause of death is CVD [5,6], which becomes more risky as kidney function declines [7]. Thus, dyslipidemia has become an important risk factor for CVD development in CKD patients. However, the typical derangements of lipoprotein metabolism in patients with CKD differ from those in the general population. While LDL-C levels tend to stay the same, TG levels rise significantly, and HDL-C levels decline in CKD patients. Moreover, the compositions of lipoproteins in CKD patients also change, including increased levels of apolipoprotein B (apoB) and lipoprotein(a) (Lp(a)) [4,8,9].

The risk of CVD in CKD patients arises from both atherosclerotic cardiovascular disease (ASCVD) and non-atherosclerotic CVD, including fluid overload leading to heart failure and arrythmias caused by hyperkalemia [10]. However, in the early and moderate stages of CKD, ASCVD accounts for the majority of CV events; by contrast, the non-atherosclerotic CVD becomes more significant until closer to the pre-ESKD stage [7,10]. Patients with early and moderate stages of CKD are more likely to die of CVD than progress to end-stage renal disease (ESRD) requiring dialysis [4,6,11]. Thus, treatments and interventions to decrease CV events in this population are crucial, because most patients with CKD experience a cardiac event before reaching ESRD. In the general population, the most effective way to lower the risk of ASCVD is by reducing low-density lipoprotein cholesterol (LDL-C) levels with statin [12,13]. Even though many studies have demonstrated that higher triglyceride (TG) levels can increase the risks of ASCVD [14,15], it remains uncertain whether using fibrate treatments to lower TG levels also reduces the risks of ASCVD. Though prior relevant studies regarding fibrate treatment revealed conflicting results, a growing body of evidence demonstrates that fibrate treatment is less effective in the general population when compared to statin treatment [16,17]. In addition, studies regarding concurrent fibrate treatment with statin are even fewer. The PROMINENT trial, a recent randomized controlled trial (RCT), however, disclosed that combining fibrate with statins does not provide more benefits than using statin treatment alone [18], we believe it is worth re-evaluating the potential advantages of concurrent fibrate treatment with statin for CKD patients for three reasons. First, the PROMINENT trial did not analyze combining fibrate with statins in the subgroup of patients with CKD. Second, as mentioned before, the typical abnormalities of lipoprotein metabolism in patients with CKD differ from those in the general population. The dyslipidemia in CKD is largely presented with increased triglyceride levels, decreased HDL-C levels, and varying levels of LDL-C [8,9,19]. Consequently, combining statin treatment with fibrate, which can lower TG levels and elevate HDL-C levels, might be a better option for CKD patients compared to the general population. Third, the post hoc analysis of the FIELD study and a meta-analysis indicated that the protective benefits of fenofibrate against CV events are more noticeable in a subgroup of patients with moderate CKD. However, the studies did not analyze the effects of combining fibrate with statin treatment in CKD patients. Moreover, the reliability of their findings was limited by their small sample size [20,21].

As a result, this study is aimed to evaluate whether concurrent fibrate use can reduce CV risks in moderate CKD (stage 3 CKD, CKD 3) patients undergoing statin therapy. According to previous studies, the concurrent use of multiple lipid-lowering agents may influence the effects of fibrate [17]; this study enrolled patients already under statin therapy but without any other type of lipid-lowering agents to compare CV outcomes between fibrate-users (fibrate plus statin) and non-users (statin treatment alone) in moderate CKD patients.

## 2. Materials and Methods

### 2.1. Data Source

This project was performed based on the Chang Gung Research Database (CGRD), which covers the Chang Gung Memorial Hospital system. This system includes 4 tertiary medical centers and 3 teaching hospitals across different regions, and is the largest medical network in Taiwan, accounting for approximately 10% of all medical services rendered in Taiwan. The CGRD has de-identified medical data pertaining to patients’ medication prescriptions, outpatient visits, inpatients orders, procedure interventions, laboratory data, and examination reports [22]. In contrast to other medical research databases, such as Taiwan’s National Healthcare Insurance Research Database, which frequently lacks detailed laboratory data and examination reports, the easy access to the laboratory data and examination reports is the major advantage of the CGRD, allowing researchers to perform in-depth analyses and avoid bias from incomplete data. Data in the CGRD that may be linked to specific patients or medical providers were encrypted and scrambled before use in this study to protect patient privacy. Thus, the requirement for written informed consent was waived. This study was approved by the Institutional Review Board of the Chang Gung Medical Foundation (approval number: 201900840B0).

### 2.2. Patient Selection and Study Design

**Figure 1:**
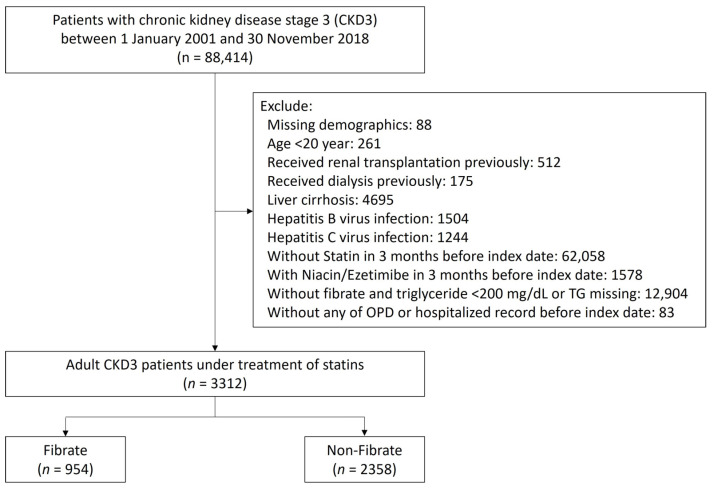
Patient inclusion–exclusion flowchart.

This study aims to determine the role of fibrate in CKD 3 patients with concurrent statin treatment. The diagnosis of stage 3 CKD was indicated by 2 consecutive records of estimated glomerular filtration rate (eGFR) between 30 and 60 mL/min/1.73 m^2^ at least 3 months apart, and the second date of eGFR was defined as the index date. The flow of patient selection is depicted in Figure 1. We focused on comparing outcomes between patients with fibrate and statin combined and patients with statin alone. Thus, patients without receiving statins were excluded. To reduce interference from the other effects of lipid-lowering agents, patients who received other lipid-lowering agents, such as ezetimibe and niacin, in the 3 months preceding the index date, were excluded as well, although excluding these patients reduced the overall number of participants and may have potentially decreased the statistical power of our study. Patients with normal TG levels (TG < 200 mg/dL) in the non-fibrate group were excluded to reduce confounding by indication. We focused on adult CKD patients, so patients under the age of 20 were excluded.

In addition, the following patients were excluded: (1) patients with missing data on demographic characteristics; (2) patients receiving kidney transplantations or any type of dialysis before the index date; and (3) patients with liver dysfunction, including hepatitis, virus infection, or liver cirrhosis. The eligible patients with stage 3 CKD treated with statin were assigned to one of two study groups: a fibrate group comprising patients treated with fibrate, and a non-fibrate group comprising patients not treated with fibrate. Patients were assigned to either group according to their prescription (fibrate or non-fibrate) within 3 months prior to the index date.

### 2.3. Covariates

We adjusted for the following demographic and clinical characteristics: age, sex, body mass index (BMI), primary renal diseases, comorbidities, medications, laboratory examination results at baseline, and numbers of admissions in the previous year before the index date. The comorbidities in question were hypertension, diabetes mellitus (DM), atrial fibrillation, peripheral artery disease, dementia, heart failure, myocardial infarction, and stroke. The ICD-9-CM and ICD-10-CM codes for comorbidities in this study are listed in Supplementary Materials (Table S1). The primary renal diseases in question were hypertensive nephropathy, DM nephropathy, chronic glomerulonephritis (e.g., lupus nephritis, IgA nephropathy, and focal segmental glomerulosclerosis), and other forms of renal disease (e.g., obstructive nephropathy and interstitial nephritis). Comorbidities and primary disease for CKD were indicated by the presence of at least 2 outpatient visits or 1 inpatient stay reported in the year prior to the index date. Baseline laboratory examination results, including the estimated glomerular filtration rate (eGFR) and levels of glycohemoglobin (HbA1c), proteinuria, serum creatinine, blood urine nitrogen, sodium, potassium, and hemoglobin were obtained using the most recent record within 3 months preceding the index date. To avoid lipid profile data obtained prior to the initiation of fibrate treatment, the latest lipid profile data during the first 3 months after the index date were used; these data pertained to the levels of triglyceride, LDL-C, HDL-C, and total cholesterol. Finally, concomitant medications were identified on the basis of prescriptions within 90 days prior to the index date.

### 2.4. Outcomes Definition

The primary outcome of this study was MACCEs, defined as a composite of AMI, CV death, and ischemic stroke. The secondary outcome was all-cause mortality. The outcomes were identified according to the medical records in the CGRD. All-cause mortality and CV death were identified based on documented mortality in the CGRD. The occurrence of AMI and ischemic stroke was ascertained in the inpatient setting. The follow-up duration was from the index date to date of death, the first occurrence of any study outcome independently, the 5th year of follow-up, or until the end date of the study period (30 November 2018), whichever came first.

### 2.5. Statistical Analysis

There was substantial difference in the baseline characteristics between the study groups (fibrate vs. non-fibrate), which may induce selection bias. Therefore, we created an adjusted cohort using inverse probability treatment weighting (IPTW) with average treatment effect based on the propensity score to balance the baseline data between the two groups. We estimated the propensity score using the generalized boosted model (GBM) with 100,000 regression trees; it is known that the performance of propensity score analysis is better in machine learning approaches than in conventional methods (e.g., logistic regression) [23]. All of the baseline data (listed in Table 1) were included in the propensity score calculation, except the follow-up duration was replaced with the index date. The balance between groups before and after IPTW was assessed using standardized difference (STD) and an absolute value of <0.2 indicated a non-substantial difference between groups. Single imputation using an expectation–maximization algorithm was employed to account for the substantial number of missing values in the continuous baseline data. All outcome comparisons were made in the complete imputed data and IPTW-adjusted cohort.

The risk of fatal outcomes between the fibrate and non-fibrate groups was compared using the Cox proportional hazard model. The incidence of other time-to-event outcomes between groups was compared using the Fine and Gray subdistribution hazard model, which considered all-cause mortality during follow-up a competing risk. Further, a subgroup analysis was conducted on MACCEs stratified by the subgroup variables: age (<65 vs. ≥65 years), sex, hypertension, DM, CKD stage (3a vs. 3b), LDL-C level, and under high-potency statin or not. Lastly, we compared the risk of MACCEs in patients who took fibrate and in patients who did not take fibrate with different triglyceride levels and HDL-C levels. The analysis was adjusted for all the covariates (listed in Table 1). Due to the existence of potential imbalance in some covariates (i.e., uric acid, use of oral hypoglycemic agents (OHAs) and antiplatelet agents; absolute STD values of >0.2) between groups even after IPTW, additional adjustments were made in the abovementioned regression models. Analyses were performed using SAS software, version 9.4 (SAS Institute, Cary, NC, USA). A two-sided *p* value of <0.05 was considered significant.

## 3. Results

### 3.1. Patient Characteristics

Figure 1 illustrates the cohort profile. We initially enrolled 3312 adult patients with a diagnosis of stage 3 CKD who received statin treatment within the 3 months preceding the index date. Of these patients, 954 patients were assigned to the fibrate group according to the combined use of fibrate and statin within 3 months preceding the index date, and 2358 patients were assigned to the non-fibrate group because their TG levels > 200 mg/dL and did not receive fibrate treatment. In fibrate group, 92.4% enrollees received Fenofibrate and only 7.6% enrollees received Gemfibrozil. The patients’ demographic and clinical characteristics are shown in Table 1. The mean TG levels for the fibrate group and non-fibrate group were 310.6 and 314.9 mg/dL, respectively; the mean LDL-C levels for the fibrate group and non-fibrate group were 122.9 and 133.3 mg/dL, respectively; and the mean HDL-C levels for the fibrate group and non-fibrate group were 40.4 and 40.2 mg/dL, respectively. We balanced the covariates between the groups by using IPTW. Before IPTW, the fibrate group had a higher prevalence of lower proteinuria and had fewer prescriptions of angiotensin converting enzyme inhibitors/angiotensin II receptor blockers and nitrates compared with those in the non-fibrate group (the absolute standard deviation (STD) values >0.2). After IPTW, all absolute STD values were less than 0.2, and most were less than 0.1, which indicates covariate balance (Table 1).

### 3.2. Five-Year Follow-Up Outcomes

**Figure 2:**
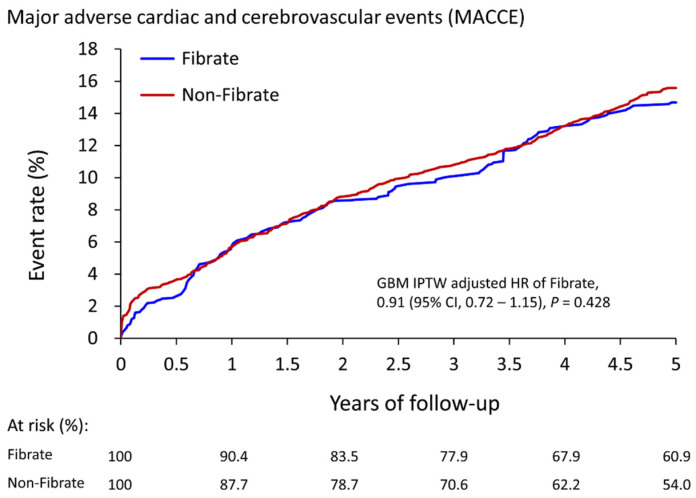
The cumulative event rate for major adverse cardiac and cerebrovascular events in patients with and without the use of fibrate (fibrate plus statin vs. statin alone) in the IPTW-adjusted cohort. IPTW, inverse probability of treatment weighting; CI, confidence interval.

After five-year follow-up, Table 2 shows that the fibrate group exhibited only a significantly lower incidence of AMI (4.4% vs. 5.4%, HR: 0.77, 95% CI: 0.61 to 0.98). The risk of MACCEs (14.7% vs. 15.6%, HR: 0.91, 95% CI: 0.72 to 1.15), all-cause mortality (5.7% vs. 6.1%, HR: 0.91, 95% CI: 0.63 to 1.30), ischemic stroke (9.2% vs. 8.9%, HR: 1.02, 95% CI: 0.85 to 1.22), and CV death (3.2% vs. 3.8%, HR: 0.81, 95% CI: 0.50 to 1.29) did not significantly differ between the two groups. The cumulative event rates of MACCEs are illustrated in Figure 2.

### 3.3. Subgroup Analysis

**Figure 3:**
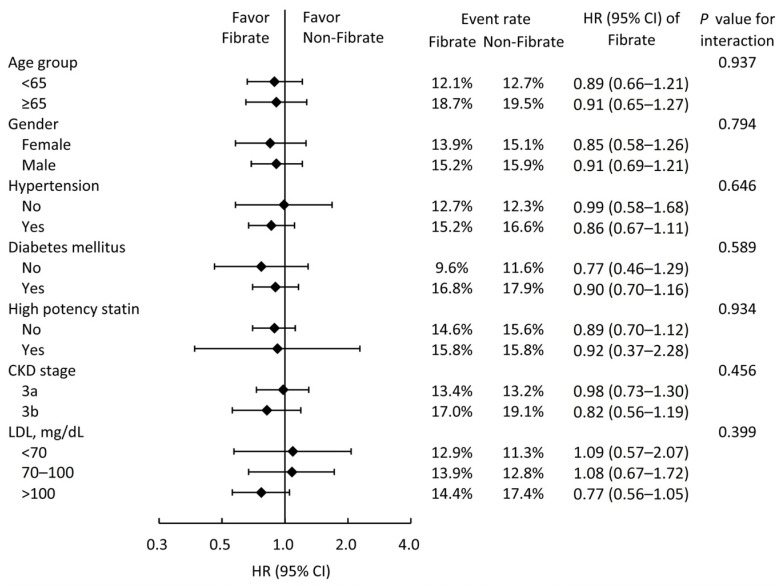
Subgroup analysis of major adverse cardiac and cerebrovascular events stratified by pre-specified baseline characteristics in the IPTW-adjusted cohort. IPTW, inverse probability of treatment weighting; HR, hazard ratio; CI, confidence interval; CKD, chronic kidney disease; LDL, low-density lipoprotein cholesterol.

We conducted subgroup analyses for MACCEs (Figure 3) to further investigate whether clinical conditions influenced the relationship between fibrate usage and primary outcomes. The results indicated that fibrate offers no supplementary benefits across all subgroups. Interestingly, the effect of fibrate on the reduction in MACCEs seems to be more predominant in the subgroup with higher LDL levels, although the results were not statistically significant.

**Figure 4:**
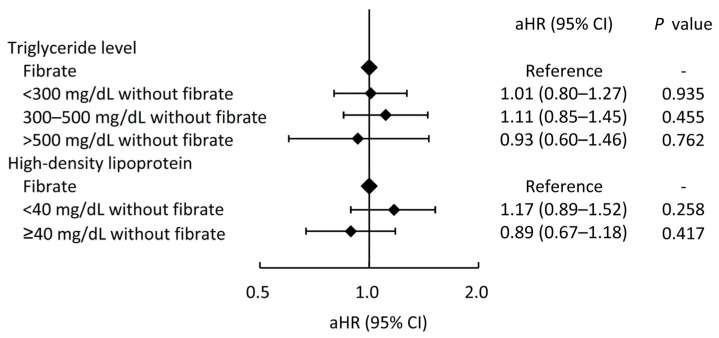
The risks of major adverse cardiac and cerebrovascular events across different triglyceride and high-density lipoprotein levels in patients who did not take fibrate compared those who took fibrate (the reference category). aHR, adjusted hazard ratio; CI, confidence interval.

In addition, we also compared whether the fibrate usage is more strongly associated with a lower risk of MACCEs in patients with higher TG levels or with lower HDL-C levels (the fibrate group was taken as a reference group), as shown in Figure 4. For patients treated with statin alone, the MACCEs risks did not significantly change across patients with different TG or HDL-C levels compared to the patients with concurrent fibrate and statin therapy. On the other hand, the different TG levels in the fibrate group also had no apparent impact on the CV outcomes compared to the non-fibrate group (Supplemental Table S2).

## 4. Discussion

In this relatively large-scale observation cohort study, among moderate CKD patients under statin treatment, fibrate reduced the risk of acute myocardial infarction (AMI) (4.4% vs. 5.4%, HR: 0.77, 95% CI: 0.61 to 0.98) but did not significantly reduce the risk of MACCEs and all-cause mortality. In subgroup analyses, the combined effect of fibrate and statin therapy on decreasing MACCEs appeared more pronounced in patients with higher LDL levels, although the results lacked statistical significance.

Fibrate, a peroxisome proliferator-activated receptor alpha agonist, effectively reduces TG levels and increases HDL-C levels [17]. It also modulates several crucial lipoproteins metabolism, especially decreasing the formation of apolipoproteins C-III [24] that inhibit lipolysis, and increases the production of apoA-I and apoA-II [25], which are the major lipoproteins of HDL-C. In the general population, most previous large-scale RCTs have shown limited or no benefits of fibrate in preventing CV events [17]. Furthermore, combining fibrate with statin could not provide additional benefits in the general population compared with those with statin treatment alone when it came to outcomes of CV events [18,26]. In patients with CKD and marked proteinuria, alterations in metabolic processes lead to hypertriglyceridemia and low HDL-C levels, which become the hallmarks of dyslipidemia [27]. However, the increase in the levels of LDL still act as crucial contributing factors in inducing atherosclerosis, while HDLs act as protective factors [28]. These metabolic alterations, which might influence the role of fibrate in CKD patients and warrant further investigation, could potentially elucidate the subgroup analyses with different LDL-C levels, suggesting that the CV beneficial effects of fibrate treatment appear less pronounced in patients with lower LDL-C levels.

In this cohort study, concurrent fibrate and statin treatment did not reduce the risk of MACCEs and all-cause mortality, and the result remained consistent in the subgroup analysis with varying TG levels. While an observational study cannot fully elucidate the detailed mechanism behind it, we speculate that fibrate and statin may share a common pathway in the prevention of CV events. Concurrent use of fibrates with statins may lead to a limited effect, possibly due to the blocking of this shared pathway by statins. One possible mechanism involves the role of small dense LDL-C (sdLDL-C), which is known to increase along with CKD progression and is more atherogenic than normal LDL-C [29,30]. Previous research has also proven that both statin and fibrate can decrease the ratio of sdLDL-C [31,32]. However, the combination of statins and fibrates may not further decrease sdLDL-C to a lower level, thus limiting additional benefits in CV protection. This mechanism might explain the results of our study. Another potential mechanism to explain the results of our study is the influence of the catabolic rate of apolipoprotein B (apoB). ApoB plays a fundamental role in the atherosclerotic process [33], and in patients with CKD, the catabolic rates of both VLDL-apoB and LDL-apoB are known to decrease [34,35]. Previous research has demonstrated that the use of fibrates can increase the catabolic rate of VLDL-apoB exclusively, whereas statin use can elevate the catabolic rates of both LDL-apoB and VLDL-apoB [36]. Therefore, the use of fibrates may confer cardiovascular protective effects by reducing VLDL-apoB in CKD patients. However, this effect may be attenuated when statins are concurrently administered, as statins not only reduce VLDL-apoB but also LDL-apoB. Further research with more detailed lipid profiles, including sdLDL-C levels and apoB, in CKD patients is necessary to validate our speculations.

Interestingly, although concurrent fibrate and statin treatment did not reduce the risk of MACCEs and all-cause mortality, it reduced the risk of AMI. In the FIELD study, which indicated similar results, fibrate also reduced total cardiovascular events but did not reduce the risk of the primary outcome of coronary events [17]. A possible hypothesis is that fibrate mainly reduces non-fatal myocardial infarctions; thus, it is not associated with MACCEs and all-cause mortality. However, further investigation is still needed to validate this hypothesis. In summary, the CV protective benefit of combining fibrate with statin therapy is not significant among CKD patients. However, according to our results, among patients with high risks of AMI or coronary artery events, the role of combined fibrate and statin is still worthy of investigation. Nonetheless, the potential side effects of fibrates and statin combination in CKD patients should be taken into consideration in this situation. The concomitant use of fibrates and statins can increase the risk of muscle injury, including rhabdomyolysis [37]. Gemfibrozil increases the plasma levels in all the statins except for fluvastatin, and thus increases the pre-disposition for rhabdomyolysis. This effect is not seen with fenofibrate and is not related to cytochrome P450 metabolism, but is due to the inhibition of the glucuronidation pathway involved in the metabolism of statins [38]. Therefore, fenofibrate is the preferred fibrate in patients who require combined therapy in both the general population and CKD patients [38,39]. Indeed, in the fibrate group of our study, 92.4% enrollees received Fenofibrate and only 7.6% enrollees received Gemfibrozil. Additionally, in a previous review article, women are at higher risk of non-adherence to statin treatment and are more pre-disposed to discontinue treatment because of side effects [40]. In this study, fibrates were found to reduce the risk of AMI. For patients unable to tolerate the complications of statins or unwilling to undergo statin treatment, treating hypertriglyceridemia with fibrates may still play a role in cardiovascular prevention.

There are numerous novel molecules that have been introduced in the treatment of diabetes mellitus, dyslipidemia, and cardiovascular disease, including glucagon-like peptide 1 (GLP-1) agonist, and proprotein convertase subtilisin-kexin type 9 (PCSK9) inhibitors [41]. However, there is still a lack of investigation into the interaction between these novel molecules and fibrate treatment. Further research is required to determine how new agents, such as PCSK9 enzyme inhibitors, interact with fibrates.

This study has two strengths: First, this study is the first study to explore the role of fibrate treatment in CKD patients undergoing statin treatment. Second, the sample size of this study was relatively large, with a total of 3312 patients (954 fibrate-users) with moderate CKD under statin treatment. Thus, the findings of this study are relatively robust and generalizable. However, this study has several limitations to acknowledge. First, the observational design of this study may have inherent bias that could not be simply eliminated by using propensity score weighting. Second, this study used data from a database, which does not guarantee the long-term compliance of fibrate and statin. Third, because we intended to include more patients into the fibrate group, we did not exclude patients with TG levels >200 mg/dL in the fibrate group. Thus, the difference in mean TG levels between the fibrate group and non-fibrate group was low. However, we additionally investigated the influence of different TG levels in the subgroup analysis. Fourth, because the users of Gemfibrozil are many fewer than fenofibrate in Taiwan (only 7.4% patients received Gemfibrozil in fibrate group), it is difficult to further evaluate the influence of different kinds of fibrate in this observational cohort study. Finally, the study population was from the Chang Gung Research Database (CGRD), accounting for approximately 10% of all medical services rendered in Taiwan. The results can be applied to the CKD population in Taiwan, although there may be limitations in generalizing the findings to different races or broader CKD populations due to a lack of diverse ethnicities and a limited number of cases.

## 5. Conclusions

Among CKD3 patients under statin treatment, the risk of MACCEs and all-cause mortality did not significantly differ between the fibrate group and the non-fibrate group. Therefore, the combination of fibrate and statin did not provide additional CV protection compared to statin therapy alone. Nonetheless, concurrent use of fibrate with statin seems to have some benefits in reducing the risk of AMI, though the mechanism is not well elucidated. This study firstly provides evidence that, among moderate CKD patients already under statin treatment, additionally treatment of hypertriglyceridemia with fibrate may not be necessary for the prevention of CV events. For patients unable to tolerate the complications of statins, further investigation into the role of treating hypertriglyceridemia with fibrates alone in cardiovascular prevention is warranted.

## Figures and Tables

**Table 1:** Baseline characteristics of the patients with and without use of fibrate (fibrate plus statin vs. statin alone).

		Before GBM IPTW					After GBM IPTW		
Variables		Available Number	Total (*n* = 3312)	Fibrate (*n* = 954)	Non-Fibrate (*n* = 2358)	MASD	Fibrate	Non-Fibrate	MASD
Age, years		3312	61.6 ± 11.9	61.4 ± 10.9	61.7 ± 12.3	−0.03	61.6 ± 11.3	61.6 ± 12.2	<0.01
Age ≥65 years		3312	1366 (41.2)	357 (37.4)	1009 (42.8)	−0.11	39.6%	42.1%	−0.05
Male		3312	1929 (58.2)	620 (65.0)	1309 (55.5)	0.19	61.0%	56.4%	0.09
Body mass index, kg/m^2^		2656	27.1 ± 4.8	27.3 ± 5.1	27.0 ± 4.7	0.07	27.1 ± 4.3	27.0 ± 4.3	0.03
Primary renal disease		3312							
	Hypertension nephropathy		740 (22.3)	190 (19.9)	550 (23.3)	−0.08	22.2%	22.7%	−0.01
	Diabetes nephropathy		1933 (58.4)	612 (64.2)	1321 (56.0)	0.17	61.2%	57.3%	0.08
	Chronic glomerulonephritis		429 (13.0)	106 (11.1)	323 (13.7)	−0.08	12.0%	13.3%	−0.04
	Others *		210 (6.3)	46 (4.8)	164 (7.0)	−0.09	4.6%	6.8%	−0.09
Comorbidities									
	Hypertension	3312	2573 (77.7)	761 (79.8)	1812 (76.8)	0.07	80.1%	76.8%	0.08
	Diabetes mellitus	3312	2163 (65.3)	686 (71.9)	1477 (62.6)	0.20	70.1%	63.8%	0.13
	Atrial fibrillation	3312	104 (3.1)	35 (3.7)	69 (2.9)	0.04	3.2%	2.8%	0.02
	Peripheral artery disease	3312	124 (3.7)	30 (3.1)	94 (4.0)	−0.05	2.9%	3.9%	−0.06
	Dementia	3312	88 (2.7)	26 (2.7)	62 (2.6)	0.01	2.5%	2.6%	−0.01
	Heart failure	3312	141 (4.3)	41 (4.3)	100 (4.2)	<0.01	3.7%	3.9%	−0.01
	Myocardial infarction	3312	219 (6.6)	68 (7.1)	151 (6.4)	0.03	5.8%	6.1%	−0.01
	Stroke	3312	368 (11.1)	97 (10.2)	271 (11.5)	−0.04	11.9%	11.7%	0.01
Admissions in the previous year		3312							
	0		2422 (73.1)	768 (80.5)	1654 (70.1)	0.24	78.3%	71.5%	0.16
	1–2		841 (25.4)	173 (18.1)	668 (28.3)	−0.24	20.5%	27.0%	−0.15
	≥3		49 (1.5)	13 (1.4)	36 (1.5)	−0.01	1.2%	1.5%	−0.02
Medication at baseline									
	High potency statin	3312	158 (4.8)	50 (5.2)	108 (4.6)	0.03	5.9%	4.7%	0.06
	ACEi/ARB	3312	1839 (55.5)	444 (46.5)	1395 (59.2)	−0.25	54.7%	57.9%	−0.06
	Beta-blockers	3312	854 (25.8)	213 (22.3)	641 (27.2)	−0.11	25.4%	27.0%	−0.04
	Calcium-channel blocker	3312	1170 (35.3)	281 (29.5)	889 (37.7)	−0.18	35.3%	37.2%	−0.04
	Spironolacton	3312	151 (4.6)	21 (2.2)	130 (5.5)	−0.17	3.0%	5.2%	−0.11
	Nitrates	3312	480 (14.5)	90 (9.4)	390 (16.5)	−0.21	10.7%	15.5%	−0.14
	Vasodilator	3312	46 (1.4)	11 (1.2)	35 (1.5)	−0.03	1.4%	1.4%	<0.01
	Thiazide	3312	362 (10.9)	96 (10.1)	266 (11.3)	−0.04	12.6%	11.1%	0.05
	Antiplatelet agents	3312	1419 (42.8)	434 (45.5)	985 (41.8)	0.08	45.4%	41.8%	0.07
	NSAIDs	3312	107 (3.2)	30 (3.1)	77 (3.3)	−0.01	3.9%	3.3%	0.04
	Steroid	3312	59 (1.8)	11 (1.2)	48 (2.0)	−0.07	1.7%	1.9%	−0.02
	Proton pump inhibitor	3312	183 (5.5)	35 (3.7)	148 (6.3)	−0.12	3.4%	5.9%	−0.12
	Insulin	3312	264 (8.0)	50 (5.2)	214 (9.1)	−0.15	6.2%	8.8%	−0.10
	OHAs	3312	1555 (47.0)	445 (46.6)	1110 (47.1)	−0.01	49.3%	47.9%	0.03
	Pentoxyfillin	3312	91 (2.7)	40 (4.2)	51 (2.2)	0.12	3.4%	2.2%	0.08
	Sodium bicarbonate	3312	9 (0.3)	1 (0.1)	8 (0.3)	−0.05	0.04%	0.36%	−0.07
Laboratory data at baseline									
	Creatinine, mg/dL	3312	1.40 ± 0.29	1.39 ± 0.28	1.41 ± 0.29	−0.06	1.40 ± 0.29	1.41 ± 0.29	−0.01
	eGFR, ml/min/1.73 m^2^	3312	47.3 ± 8.4	48.6 ± 8.2	46.8 ± 8.5	0.22	47.8 ± 8.5	47.0 ± 8.5	0.08
	Blood urine nitrogen, mg/dL	1773	24.2 ± 10.2	23.5 ± 9.3	24.4 ± 10.5	−0.09	23.5 ± 7.5	23.8 ± 8.1	−0.04
	Proteinuria group, mg/dL	1794							
	Negative or Trace (0–29)		834 (46.5)	262 (56.7)	572 (42.9)	0.28	52.1%	43.8%	0.17
	1+ or 2+ (30–299)		486 (27.1)	110 (23.8)	376 (28.2)	−0.10	24.7%	28.0%	−0.08
	3+ or 4+ (≥300)		474 (26.4)	90 (19.5)	384 (28.8)	−0.22	23.2%	28.2%	−0.11
	Potassium, mg/dL	1973	4.1 ± 0.6	4.2 ± 0.6	4.1 ± 0.6	0.15	4.2 ± 0.4	4.2 ± 0.5	0.08
	Sodium, mg/dL	1609	138.9 ± 4.0	139.2 ± 4.0	138.8 ± 4.0	0.09	138.9 ± 2.9	139.0 ± 3.0	−0.02
	HbA1C, %	2409	8.4 ± 2.2	8.1 ± 1.9	8.5 ± 2.3	−0.19	8.0 ± 1.8	8.2 ± 2.1	−0.08
	Albumin, g/dL	1026	3.6 ± 0.9	3.9 ± 0.7	3.5 ± 0.9	0.56	3.8 ± 0.5	3.7 ± 0.6	0.13
	Hemoglobin, g/dL	1884	12.7 ± 2.1	12.7 ± 2.1	12.7 ± 2.1	0.02	12.9 ± 1.6	12.9 ± 1.8	<0.01
	Uric acid, mg/dL	2024	7.5 ± 2.0	6.9 ± 1.9	7.7 ± 2.0	−0.44	7.2 ± 1.5	7.5 ± 1.6	−0.19
Lipids profile at inclusion									
	LDL, mg/dL	2956	130.2 ± 70.0	122.9 ± 66.7	133.3 ± 71.2	−0.15	123.2 ± 65.0	134.4 ± 69.2	−0.17
	HDL, mg/dL	2362	40.3 ± 10.5	40.4 ± 10.7	40.2 ± 10.4	0.01	40.1 ± 9.0	40.0 ± 8.9	0.01
	Total cholesterol, mg/dL	2689	219.6 ± 64.8	208.2 ± 58.4	224.7 ± 66.8	−0.26	216.4 ± 58.6	223.1 ± 62.7	−0.11
	Triglyceride, mg/dL	3296	313.7 ± 195.6	310.6 ± 274.1	314.9 ± 153.7	−0.02	315.7 ± 208.3	323.3 ± 169.9	−0.04
Follow-up duration, year		3312	7.0 ± 4.1	7.1 ± 3.9	6.9 ± 4.2	0.05	7.5 ± 3.9	7.0 ± 4.2	0.12

Abbreviations: MASD, maximum absolute standardized difference; GBM, generalized boosted models; IPTW, inverse probability of treatment weighting; ACEi/ARB, angiotensin-converting enzyme inhibitors/angiotensin receptor blocker; NSAIDs, non-steroidal anti-inflammatory drugs; OHAs, oral hypoglycemic agents; eGFR, estimated glomerular filtration rate; HbA1C, glycated hemoglobin; LDL, low-density lipoprotein; HDL, high-density lipoprotein. Data are presented as frequency (percentage), mean ± standard deviation or median (25th, 75th percentile); * including interstitial nephritis, obstructive nephropathy, polycystic kidney, and others.

**Table 2:** Time-to-event outcomes during the 5-year follow-up (fibrate plus statin vs. statin alone).

	Before IPTW *		After GBM IPTW #			
Outcome	Fibrate (*n* = 705)	Non-Fibrate (*n* = 2020)	Fibrate	Non-Fibrate	HR/SHR (95% CI) of Fibrate	*p* Value
All-cause death	54 (5.7)	151 (6.4)	5.7%	6.1%	0.91 (0.63–1.30)	0.591
MACCEs						
Cardiovascular death	29 (3.0)	95 (4.0)	3.2%	3.8%	0.81 (0.50–1.29)	0.365
Acute myocardial infarction	45 (4.7)	130 (5.5)	4.4%	5.4%	0.77 (0.61–0.98)	0.035
Ischemic stroke	84 (8.8)	214 (9.1)	9.2%	8.9%	1.02 (0.85–1.22)	0.831
Composite outcome $	138 (14.5)	376 (15.9)	14.7%	15.6%	0.91 (0.72–1.15)	0.428

Abbreviation: IPTW, inverse probability of treatment weighting; HR, hazard ratio; SHR, subdistribution hazard ratio; CI, confidence interval; MACCEs, major adverse cardiac and cerebrovascular events. * Data are presented as frequency (percentage). # Data are presented as percentage. $ Any of cardiovascular death, acute myocardial infarction, or ischemic stroke.

## Data Availability

The data presented in this study are available on request from the corresponding author. The data are not publicly available because the data in the CGRD can only be obtained and used inside Chang Gung Memorial Hospital.

## Supplementary Materials

The following supporting information can be downloaded at: https://www.mdpi.com/article/10.3390/jcm13010168/s1, Table S1: Disease code use in this study; Table S2: Time to event outcomes during the 5-year follow-up after GBM IPTW.
