# Supplementary material for: Can Concurrent Fibrate Use Reduce Cardiovascular Risks among Moderate Chronic Kidney Disease Patients Undergoing Statin Therapy? A Cohort Study
Source: J Clin Med. 2023 Dec 28;13(1):168. doi: 10.3390/jcm13010168 (PMC10779526; doi:10.3390/jcm13010168)
Supplement: Supplementary file 1 [file jcm-13-00168-s001.zip › jcm-2713354-supplementary.pdf]

Supplemental Table S1. Disease code use in this study

Disease	ICD-9-CM	ICD-10-CM
Liver cirrhosis	571.2x, 571.5x, 571.6x	K70.30, K74.0, K74.60, K74.69, K74.3, K74.4, K74.5
Hepatitis B virus infection	070.20, 070.22, 070.30, 070.32, V02.61	B18.0, B18.1, B16.2, B19.11, B16.9, B19.10
Hepatitis C virus infection	070.41, 070.44, 070.51, 070.54, 070.70, 070.71, V02.62	B18.2, B17.10, B17.11, B19.20, B19.21
Hypertension nephropathy	403.9x	I12
Diabetes nephropathy	250.4x	E08.2, E09.2, E10.2, E11.2, E13.2
Chronic glomerulonephritis	582.x	N03
Interstitial nephritis	583.89	N11.8, N11.9, N14, N15.8 , N15.9
Obstructive nephropathy	599.6	N13 combined with N18.3 to N18.6
Polycystic kidney disease	753.12, 753.13, 753.14	Q61.1, Q61.2, Q61.3
Hypertension	401.xx-405.xx	I10-I15, N262
Diabetes mellitus	250.xx	E08-E13
Atrial fibrillation	427.3x	I48
Peripheral arterial disease	440.xx, 441.xx, 443.xx, 444.0x, 444.8x, 447.8x, 447.9x, 093.0, 437.3, 444.22, 447.1, 557.1, 557.9, V434	I70, I71, I73, I75, I771, I790, I791, I792, I773, I779, I798, K551, K558, K559, Z958, Z959, I743, I744, I745, I748, I740, I7789
Dementia	290.xx, 294.xx	F03.90, F05, F01.50, F01.51, F04, F02.80, F02.81, F03.90, F03.91, F06.0, F06.1, F06.8, F09
Heart failure	428.xx	I50
Myocardial infarction	410.xx, 412.xx	I21-I22
Stroke	430.xx–437.xx	I60-I62, I66, I65.1, I65.0, I65.8, I65.9, I63.6, I63.8, I63.9,

Disease	ICD-9-CM	ICD-10-CM
		G45.0, G45.8, G45.1, G45.2, G46.0, G46.1, G46.2, G45.9, G45.4, G46.3, G46.4, G46.5, G46.6, G46.7, G46.8, I67.0, I67.1, I67.2, I67.4, I67.5, I67.6, I67.7, I67.9, I68.0, I68.2, I68.8
Acute myocardial infarction	410.xx	I21
Ischemic stroke	433.xx–437.xx	I66, I65.1, I65.0, I65.8, I65.9, I63.6, I63.8, I63.9, G45.0, G45.8, G45.1, G45.2, G46.0, G46.1, G46.2, G45.9, G45.4, G46.3, G46.4, G46.5, G46.6, G46.7, G46.8, I67.0, I67.1, I67.2, I67.4, I67.5, I67.6, I67.7, I67.9, I68.0, I68.2, I68.8
Renal transplantation	V42.0	Z94.0

Supplemental Table S2. Time to event outcomes during 5 years follow-up after GBM IPTW

Outcome	Fibrate & TG <200	Fibrate & TG ≥200	Non-Fibrate	HR/SHR (95% CI) of Fibrate & TG <200	*P* value	HR/SHR (95% CI) of Fibrate & TG ≥200	*P* value
MACCEs							
Cardiovascular death	3.5%	3.2%	3.8%	1.08 (0.78–1.48)	0.646	0.89 (0.67–1.18)	0.410
Acute myocardial infarction	6.0%	3.9%	5.3%	1.18 (0.90–1.54)	0.238	0.67 (0.53–0.86)	0.002
Ischemic stroke	10.0%	9.7%	8.8%	1.08 (0.89–1.31)	0.440	1.07 (0.90–1.28)	0.424
Composite outcome$	17.6%	14.9%	15.4%	1.19 (1.02–1.37)	0.024	0.93 (0.81–1.06)	0.287

Abbreviation: IPTW, inverse probability of treatment weighting; HR, hazard ratio; SHR, subdistribution hazard ratio; CI, confidence interval; MACCEs, major adverse cardiac and cerebrovascular events;

$ Any of cardiovascular death, acute myocardial infarction or ischemic stroke
